# Antioxidant, Cytotoxic, and DNA Damage Protection Activities of Endophytic Fungus *Pestalotiopsis neglecta* Isolated from *Ziziphus spina-christi* Medicinal Plant

**DOI:** 10.3390/microorganisms11010117

**Published:** 2023-01-01

**Authors:** Hibah I. Almustafa, Ramy S. Yehia

**Affiliations:** 1 Department of Biological Sciences, College of Science, King Faisal University, Al-Ahsa 31982, Saudi Arabia; 2 Department of Botany and Microbiology, Faculty of Science, Cairo University, Giza 12613, Egypt

**Keywords:** bioactive metabolites, endophytic fungi, GC-MS analysis, *Pestalotiopsis neglecta*, *Ziziphus spina-christi*

## Abstract

Fungal endophytes are friendly microorganisms that colonize plants and are important in the interactions between plants and their environment. They generate valuable secondary metabolites that are valuable to both plants and humans. Endophytic fungi with bioactivities were isolated from the leaves of the medicinal plant *Ziziphus spina-christi*. An efficient isolate was selected and identified as *Pestalotiopsis neglecta* based on nucleotide sequencing of the internal transcribed spacer region (ITS 1-5.8S-ITS 2) of the 18S rRNA gene (NCBI accession number OP529850); the 564 bp had 99 to 100% similarity with *P. neglecta* MH860161.1, AY682935.1, KP689121.1, and MG572407.1, according to the BLASTn analysis, following preliminary phytochemical and antifungal screening. The biological activities of this fungus’ crude ethyl acetate (EtOAc) extract were assessed. With an efficient radical scavenging activity against 2,2′-diphenyl-1-picrylhydrazyl and an IC_50_ value of 36.6 µg mL^−1^, *P. neglecta* extract has shown its potential as an antioxidant. Moreover, it displayed notable cytotoxic effects against MCF-7 (breast carcinoma, IC_50_ = 22.4 µg mL^−1^), HeLa (cervical carcinoma, IC_50_ = 28.9 µg mL^−1^) and HepG-2 (liver carcinoma, IC_50_ = 28.9 µg mL^−1^). At 10 µg mL^−1^, EtOAc demonstrated significant DNA protection against hydroxyl radical-induced damage. Based on FT-IR and GC-MS spectral analysis, it was detected that the EtOAc of *P. neglecta* product contains multiple bioactive functional groups. Subsequently, this validated the features of major different potent compounds; tolycaine, 1H-pyrazol, 1,3,5-trimethyl-, eugenol, 2,5-cyclohexadiene-1,4-dione, 2,6-bis(1,1-dimethyl), and bis(2-ethylhexyl) phthalate. Since these compounds are biologically relevant in various aspects, and distinct biological activities of fungal extract were acceptable in vitro, this suggests that endophytic fungus *P. neglecta* may be a viable source of bioactive natural products. This could be a good starting point for pharmaceutical applications.

## 1. Introduction

Natural sources of useful compounds that can support the creation of innovative medications include plants. As a result, the plant’s antiulcer, antioxidant, antibacterial, anti-inflammatory, analgesic, and wound healing properties have received substantial study worldwide. Nevertheless, growing the plants and extracting their bioactive metabolites take time. Thus, research into new resources, particularly endophytic fungi (endophytes) collected from medicinal plants and capable of producing plant-specific compounds, is gaining traction [1]. Endophytes are all microorganisms that penetrate the internal tissues of living plants without harming the host or causing immediate disease symptoms; this is the most comprehensive and frequently used definition [2].

Through competition for their hosts’ food sources and habitat, endophytic fungi help to defend their hosts from pathogenic microorganisms [3]. Additionally, they work to prevent infection by harmful organisms by rapidly invading its hosts and depleting nutrients that are needed for the growth of those pathogenic microbes [4]. Endophytic fungi can act as defensive systems for their hosts, defending them against animals and insects [5].

As endophytes, different fungi were recovered from the tissues of most medicinal plants, especially the leaves [6]. The emergence of numerous drug-resistant microbes necessitated the exploration for novel antimicrobials to treat human disorder. As a result of these factors, an extensive search for innovative and more effective agents to combat these disease issues is currently ongoing [7]. Several studies have shown that endophytes are important repositories of secondary metabolites with diverse biological activities such as antiviral, anticancer, antioxidant, and antimicrobial properties [8]. Secondary metabolites such as phenols, alkaloids, saponins, steroids, terpenoids, flavonoids, and glycosides are responsible for these activities [9].

*Pestalotiopsis* has received considerable interest in recent years due to the fact that different immunosuppressants, antioxidants, anticancer agents, and a wide range of chemically novel diverse metabolites have been found from this genus; some of them may be useful as future therapeutic leads for the treatment of human diseases and the control of plant diseases [10]. It is intriguing that the fungus *P. neglecta* can offer alternative approaches for finding natural products drugs that can be reliable, cost-effective, and ecologically friendly [11]. Several studies have discovered that various species produce taxol, while others afforded a variety of bioactive natural products with therapeutic value, such as polyketides and terpenoids [12,13,14].

According to Elsabea [15], the Al-Ahsa Oasis in Saudi Arabia is regarded as one of the leading agricultural regions. It is also rich in varieties known for their exceptional processing properties, as well as numerous undiscovered varieties. Many studies suggested that in addition to the oasis’s considerable economic importance, it also has a large number of therapeutic plants [16].

*Ziziphus*, also known as “Sedras,” is a prominent genus of the Rhamnaceae family that is widely distributed in the Al-Ahsa Oasis [17]. *Ziziphus* is well-known for its anticancer, antibacterial, antioxidant, hypertensive, anti-inflammatory, and liver protector [18]. Thus, the aim of this study is to isolate and identify endophytic fungus *P. neglecta* residing in the leaves of the medicinal plant, *Z. spina-christi*, as well as to demonstrate the ability to produce bioactive agents with pharmaceutical potential, which may provide a new lead in the pursuit of new biological source of drug candidates.

## 2. Materials and Methods

### 2.1. Collections of Plant Samples

In March 2022, fresh healthy leaves of *Ziziphus spina-christi* were collected from Al-Ahsa Oasis in Saudi Arabia’s Eastern region (25°20′02.9″ N 49°34′39.9″ E). The samples were rinsed with running tap water within 24 h of collection, followed by washing with double distilled deionized water to eliminate surface debris such as dust and bird waste. The cleaned leaves were divided into 1 cm × 1 cm-size segments. Surface sterilization was performed under aseptic conditions with successive washes in 70% ethanol (30 s), 4% sodium hypochlorite solution (90 s), sterilized distilled water (20 s), finally allowing it to air dry in the laminar airflow chamber [19].

### 2.2. Isolation of Endophytes

Briefly, sterilized segments were loaded onto potato dextrose agar (PDA, 4 g potato extract, 20 g dextrose, agar 20 g, and 1 L water, HiMedia, Mumbai, India) medium supplemented with 100 μg mL^−1^ streptomycin to prevent bacterial growth in order to isolate endophytic fungi. After 4–6 days at 28 °C, endophytic fungi usually start to develop hyphal filaments. The growth of fungal endophytic colonies from the segments was monitored daily in petri dishes and individual hypha tips were picked out in time and transferred to new PDA medium. This step was carried out several times until only pure fungal colonies were achieved. The initial identification of the recovered pure fungal isolates was performed based on their morphological and microscopic characteristics in accordance with manuals for standard identification [20,21,22,23]. Endophytic fungi recovered from *Z. spina-christi* leaves have been codified as YH-1–YH-26. To estimate the abundance and preference distribution of endophytic fungal species, the colonization frequency (CF) and dominance (DF) were recorded [24,25] as follows:

$$\mathrm{CF}=\frac{\mathrm{number}\;\mathrm{of}\;\mathrm{segments}\;\mathrm{colonized}\;\mathrm{by}\;\mathrm{fungi}\;}{\mathrm{total}\;\mathrm{number}\;\mathrm{of}\;\mathrm{segments}\;\mathrm{observed}}\;\times \;100,$$

$$\mathrm{DF}=\frac{\mathrm{percentage}\;\mathrm{colony}\;\mathrm{frequency}\;}{\mathrm{the}\;\mathrm{sum}\;\mathrm{of}\;\mathrm{the}\;\mathrm{percentage}\;\mathrm{of}\;\mathrm{colony}\;\mathrm{frequency}\;\mathrm{of}\;\mathrm{all}\;\mathrm{fungi}\;}\;\times \;100.$$

### 2.3. Preliminary Screening of Bioactive Properties of Fungal Isolates

The endophytic isolates employed in this investigation were among a number of fungal isolates recovered from surface-sterilized leaf fragments that had a high colonization rate.

### 2.4. Antifungal Activity

Four fungal isolates (YH-4, YH-10, YH-16, and YH-26) were evaluated for antifungal activity against pathogens (*Alternaria alternata*, *Candida albicans*, *Fusarium oxysporum*, *Botrytis cinerea*, and *Pythium ultimum*) that were kindly obtained from the Department of Botany and Microbiology, University of King Saud, using a dual culture approach [26]. Thus, pathogens were inoculated in the center of PDA plates, and representative endophytic isolates (5 mm) were seeded on three corners of PDA plates for analysis. Each experiment was carried out three times, and all plates were incubated for 5–8 days at 28 °C. Mycelial growth of the studied fungal pathogen was inhibited in the direction of an active endophytic fungus, indicating antifungal activity. The inhibition level was measured by subtracting the fungal growth radius from the distance (mm) of fungal growth in the direction of an antagonist colony. The width of inhibitory zones was classified into three categories as >10 mm (strong inhibition, +++), 2–10 mm (moderate inhibition, ++), and <2 mm (weak inhibition, +).

### 2.5. Fungal Metabolites Extraction

Each isolated endophytic fungus (YH-4, YH-10, YH-16, and YH-26) was cultured for 5–7 days at 25 °C in a 500 mL conical flask with 150 rpm shaking containing 200 mL of sterilized potato dextrose broth (PDB, dextrose 20 g L^−1^, potato extract 4 g L^−1^, Oxoid, UK) until the stationary phase was achieved. After the incubation period, the mycelial biomass that had accumulated in the flask was filtered to remove the mycelial mat using sterile Whatman No. 1 filter paper. Following that, the supernatant was vigorously shaken for 15–20 min in a separating funnel with an equal volume of ethyl acetate (EtOAc). The EtOAc phase containing metabolites was then collected in a conical flask (500 mL) and repeated twice before evaporation with a rotary evaporator (Ika, Germany). The crude extracts were then concentrated and stored at 4 °C.

### 2.6. Phytochemical Screening

The standard procedure outlined by Kokate [27] and Maobe [28] was carried out to perform preliminary phytochemical analysis on the crude extracts of endophytic fungal isolates.

Alkaloids

A total of 5 mL solution of 2 M HCl was mixed with 1 mL of fungal crude extracts and a few drops of Mayer’s reagent (3 mL of potassium iodide mixed with mercuric chloride solution). The presence of alkaloids was assumed to be indicated by turbidity or creamish precipitate.

Flavonoids

Two to three drops of 20% NaOH solution were added to 1 mL of fungal crude extract. The presence of flavonoids was indicated by yellow color.

Phenols

A total of 1 mL of fungal crude extract was mixed with 5 mL of distilled water, followed by two to three drops of 5% ferric chloride solution. Dark green color indicated the presence of phenols.

Saponins

In a test tube, 1 mL of fungal extract was rapidly shaken for 10 min. Saponin emulsion was fairly stable.

Steroids

A total of 3 mL of chloroform were added to 1 mL of the fungal extract and filtered. A few drops of H_2_SO_4_ were added carefully. The presence of steroids was revealed by a blue-green ring at the interface.

Tannins

The presence of tannins was detected by a bluish black color that resulted from the addition of FeCl_3_ to the fungal crude extract.

Terpenoids

A total of 1 mL of crude fungal extract was added to 2 mL of chloroform, which was then boiled with 3 mL of H_2_SO_4_. A reddish brown color developed, indicating the presence of terpenoids.

### 2.7. Large-Scale Cultivation

Pure fungal isolate was inoculated into a 1000 mL Erlenmeyer flask with sterilized PDB after the efficient endophyte (YH-26) was chosen based on the findings of phytochemical screening and in vitro inhibitory efficacy against various pathogens. For four weeks, the cultivation was incubated at 28 ± 2 °C. The cultures were extracted after the incubation period using an equivalent volume of EtOAc. After the mixture was filtered using a Buchner funnel under vacuum, it was repeatedly extracted with EtOAc until exhaustion. To obtain the crude fungal extract, the EtOAc extract was concentrated using a rotatory evaporator (Buchi, Switzerland). After that, the crude extract was diluted with Dimethyl sulphoxide (DMSO, 100 μg μL^−1^). It was a syringe used for gas chromatography-mass spectrometry and biological activities examination.

### 2.8. Genotypic Identification

Sequence analysis of the ITS1-5.8S-ITS2 and ITS regions of the ribosomal ribonucleic acid (RNA) gene was carried out to corroborate the morphological identification of the YH-26 isolate. Using SDS extraction protocol outlined by Plaza [29], total fungal genomic DNA was directly recovered from mycelium that was actively growing in potato dextrose broth (PDB, Oxoid, UK). The isolated DNA was exposed to the polymerase chain reaction (PCR, Applied Biosystem, USA) utilizing primers ITS1: TCCGTAGGTGAACCTGCGG and ITS4: TCCTCCGCTTGATATGC [30]. Following that, the BigDye Deoxy Terminator cycle-sequencing kit (Applied Biosystems, Darmstadt, Germany) was applied to purify the amplified product and sequence it in an automated DNA sequencer (ABI PRISM 3700). The acquired fungal sequence was matched with previously species sequences in NCBI GenBank (http://www.ncbi.nlm.nih.gov.blast (accessed on 12 January 2022)). The identification of endophytic fungus was carried out based on the homology of amplified sequences to those in the GenBank database. The relevant isolate’s accession number was acquired. Using MEGA version 7.0, the phylogenetic analysis of sequences was constructed.

### 2.9. Biological Activity

Scavenging 2,2-diphenyl-1-picrylhydrazyl (DPPH) free radicals assay

The antioxidant activity of the EtOAc extract was investigated using the DPPH (Sigma-Aldrich, St. Louis, MO, USA) radical scavenging assay. The procedure was adopted from Yehia [31] with slight modifications, and involved preparing (0.1 mM) by dissolving 1.9 mg of DPPH in 100 mL of ethanol, keeping in the dark for 1 h, and then adding 1 mL of this solution to 2 mL of fungal extract at different doses (5, 10, 25, 50, 100 µg mL^−1^). The mixture was shaken and allowed to stand at room temperature for 60 min before the absorbance of the resulting solution was measured at 517 nm (Spectrophotometer Plus, Japan). As a control, ethanol was utilized instead of DPPH solution. To obtain a blank, distilled water was used instead of a sample. A positive control experiment was used; Vitamin c (Vc, Merck, India). The ability of DPPH radicals (DC) to scavenge free radicals was calculated using the following equation:

$$\mathrm{DC}=1-\frac{\mathrm{As}-\mathrm{Ab}}{A0}\;\times \;100$$

where, A_s_, A_b_ and A_0_ denote the absorbance values of the samples, background, and blank solution, respectively.

The IC_50_ (concentration of test sample that reduces the absorption of DPPH solution by 50%, µg mL^−1^) was calculated from the curve of the dependence of DC (%). The results of each experiment were performed in triplicate (*n* = 3) and presented as mean values.

### 2.10. Cytotoxic Assay

As described by Mahnashi et al. [32], the MTT test was performed to evaluate the impact of EtOAc crude extract on the cell viability of three tumor cell lines (Shanghai Bioleaf Technology Co. Ltd., Shanghai, China); liver carcinoma (HepG-2), cervical carcinoma (HeLa), and breast carcinoma (MCF-7) in order to quantify the reduction in MTT in living cells (yellow to purple). In 100 µL of Dulbecco’s Modified Eagle Medium (DMEM; Life Technologies, Gaithersburg, MD, USA), augmented with 100 U mL^−1^ penicillin and 100 µg mL^−1^ streptomycin (Sigma-Aldrich, St. Louis, MO, USA), cell lines were seeded at a cell density of 5000 per well. The seeded cell lines were subjected to various doses of 5, 10, 25, 50, 100 µg mL^−1^ of EtOAc extract, whereas the control cells received just the medium. Two days at 37 °C in a humidified environment with 5% CO_2_ (Thermo, Forma 370) were spent incubating the plates. Following the incubation, at a concentration of 5 mg mL^−1^, 10 µL of [MTT 3-(4,5-dimethylthiazol-2-yl)–2,5-diphenyltetrazolium bromide] was added to each well and incubated for additional 1 h at 37 °C. The purple formazan crystals were dissolved in each well by adding 100 µL of DMSO, and the results were then quantified in a microplate reader (Infinite 200 Pro, Tecan, Switzerland) after 10 min of shaking in an automated shaker in the dark. The percentage of cell viability was recorded by dividing the absorbance 570 nm of sample by the control’s absorbance and multiplying by 100. The IC_50_ values (EtOAc crude extract concentration that inhibits cell viability by 50%) were also determined. All experiments were carried out in triplicate (*n* = 3).

### 2.11. DNA Protection Assay

With minor modifications to the protocol described by Ghanta [33], the capacity of endophytic fungal EtOAc extract to shield oxidative λ-DNA (Merk, India) from destructive effects of hydroxyl radicals produced by Fenton’s reagent was evaluated. Before adding the loading dye, 0.5 μg of λ-DNA (3 μL) was incubated with 10 μL of Fenton’s reagent (25 mM H_2_O_2_ in Tris buffer 10 mM, 1 mM FeSO4, pH 7.4) at a final reaction volume of 30 μL for 45 min at 37 °C with or without different extract concentrations (5 μL: 0.1, 1.0 and 10 μg mL^−1^). On 1% agarose gel electrophoresis, the relative differences between oxidized and native DNA were analyzed. A Gel Doc system (Bio-Rad, Hercules, CA, USA) was used to visualize the gel and assess the band intensity. The positive control employed was Quercetin.

### 2.12. FT-IR Analysis

EtOAc crude extract underwent Fourier-transform infrared analysis using a Nicolet 6700 spectrometer (Thermo Scientific, Waltham, MA, USA). A Potassium Bromide-KBr pellet was prepared by mixing 1 mg of dried EtOAc fungal extract with 10 mg anhydrous KBr powder. The spectra with a scan range of 4000–500 cm^−1^. The resulting spectra were analyzed and recorded.

### 2.13. Identification of Bioactive Constituents by GC-MS

To identify the bioactive compounds, EtOAc crude extract was analyzed using gas chromatography-mass spectrometry (GC-MS) on an Agilent 7820A Gas Chromatography (GC, Agilent Technologies Inc., Santa Clara, CA, USA) equipped to Mass Spectrometer (MS, ISQ Single Quadrupole, Waltham, MA, USA). The auto sampler AS1300 was used to inject 1µL of fungal extract into the chromatography column (30 m × 0.25 mm × 0.25 µm). The instrument’s temperature was initially held at 40 °C for 2 min, then raised to 260 °C. Subsequently, it was gradually increased to 290 °C for 2 min. Helium of the highest purity was used as a carrier at a rate of 1 mL min^−1^. A total of 70 eV served as ionization voltage. 45–1000 m/z was adopted as the mass spectral scan range. In order to identify the bioactive compounds, their mass spectra and retention times (RT) were calculated and compared to data from the National Institute of Standards and Technology library (NIST14, US).

### 2.14. Statistical Analysis

Three (*n* = 3) replicates of the results were used to calculate the mean and standard deviation (SD). Using the statistical software SPSS v21.0, the data were analyzed (SPSS, Inc., Chicago, IL, USA).

## 3. Results

### 3.1. Isolation and Morphological Identification of Endophytic Fungi

In the Eastern region of Saudi Arabia, this is likely the first report to characterize the endophytic fungi that colonize *Z. spina leaves*. At first, morphological characteristics, microscopic examinations, and spore morphology were applied to identify isolates. All of the isolated endophytic fungi, which were successfully recovered from 380 leaf segments, were assigned to 11 genera. A total of 191 endophytic fungal isolates with a total colonization frequency of 50.3% were obtained. These fungi were morphotypically recognized as belonging to 26 different species, as shown in (Table 1). Trichocomaceae (27.3%), Sporocadaceae (17.9%), Chaetomiaceae (11.9%), Nectriaceae (10%), Hypocreaceae (9.3%), Davidiellaceae (8.4%), Xylariaceae (5.4%), Periconia (4.2%) and Glomerellaceae (4.8%) were the nine families of the fungal class Ascomycota, which constituted the majority of endophytic fungi. From this point, *Pestalotiopsis neglecta*, 7.6%; *Chaetomium globosum*, 5%; *Acremonium cyanophagus*, 4.7%; and *Aspergillus flavus*, 4.2% were the predominant species that occurred with a high relative frequency. As a result, we decided to choose these isolates for subsequent preliminary screening assays.

### 3.2. Preliminary Screening for Antifungal Activity

In order to investigate the potential antagonistic activity against the pathogenic fungi *Alt. alternata*, *C. albicans*, *F. oxysporum*, *B. cinerea*, and *P. ultimum*, four major fungal isolates, *P. neglecta*, *C. globosum*, *A. cyanophagus*, and *A. flavus* were evaluated using the dual culture method (Table 2). In this study, the results were recorded as strong inhibition (+++), moderate inhibition (++), weak inhibition (+), or no inhibition (−) against microbial pathogens. Our findings demonstrated that at least two of the tested pathogens were inhibited by all endophytic fungi. Fortunately, only one isolate, *P. neglecta*, inhibited the growth of all pathogens, followed by *C. globosum*, *A. flavus* and *A. cyanophagus*. By a thorough analysis of the inhibitory results, *Alt. alternata*, *F. oxysporum*, *B. cinerea* and *P. ultimum* were all strongly inhibited by *P. neglecta* while *C. albicans* was only moderately inhibited. In addition, *C. globosum* inhibited *B. cinerea* with strong inhibition, while *Alt. alternata* with moderate inhibition, and *C. albicans*, *F. oxysporum* and *P. ultimum* with weak inhibition. Further, strong inhibition was shown by *A. flavus* for *B. cinerea*, whereas moderate inhibition for *C. albicans* and *P. ultimum*, were recorded and weak inhibition for *Alt. alternata* but not active for *F. oxysporum*. On the other hand, strong inhibition against *F. oxysporum*, moderate inhibition against *P. ultimum*, and weak inhibition against *C. albicans,* while there was no inhibition against *Alt. alternata* and *B. cinerea* were doccumented by *A. cyanophagus*. In conclusion, it was found that the *P. neglecta* isolate demonstrated remarkable antifungal capacity against microbial pathogens by comparing the inhibitory impact caused by other isolates.

### 3.3. Production and Extraction of Secondary Metabolites 

*P. neglecta*, *C. globosum*, *A. flavus* and *A. cyanophagus* were cultivated in PDB medium and extracted with EtOAc yielding crude secondary metabolites of 92.4 mg, 30.6 mg, 8.7 mg and 16.5 mg, respectively. The metabolites produced by these EtOAc crude extracts were investigated in order to pinpoint the relevant fungal isolate to be used in subsequent study.

### 3.4. Qualitative Screening for Secondary Metabolites

Fungal crude extracts were subjected to chemical analysis to evaluate whether chemical scaffolds such as phenols, flavonoids, terpenoids, alkaloids, tannins, steroids, and saponins were present or absent (Table 3). Alkaloids, saponins, steroids, and terpenoids were the only phytocomponents present in the EtOAc extract of *A. cyanophagus*, whereas phenols, flavonoids, tannins, and steroids were detected in *A. flavus*. *C. globosum*, on the other hand, displayed only phenols, terpenoids, alkaloids, tannins. In contrast, *P. neglecta* revealed all bioactive metabolites. *P. neglecta*, as previously mentioned, was one of the fungal endophytes that showed potent antifungal activity as well as the capacity to create a variety of active metabolites. Therefore, the strategies will be constructed to utilize this endophytic fungus for recovery of bioactive compounds and analyze their biological properties.

### 3.5. Molecular Identification of Endophytic Fungus

**Figure 1:**
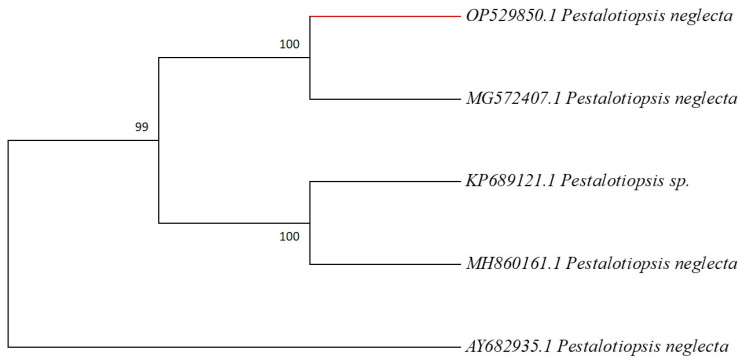
Phylogenetic tree based on the 5.8S-rRNA-ITS regions between the endophytic fungus *P. neglecta* and published data and evolutionary distances computed using the Maximum Likelihood method.

Due to their robust metabolite production and antifungal activity, genotypic approaches were used in addition to morphological characterization to corroborate the identification of the most promising endophytic fungal isolate, YH-11—*P. neglecta*. Based on the partial sequence of 18S ribosomal RNA gene; internal transcribed spacer 1, 5.8S ribosomal RNA gene, and internal transcribed spacer 2, complete sequence and the partial sequence of 28S ribosomal RNA gene, the fungal sequence was submitted to NCBI GenBank database with accession number OP529850.1. For final identification, the sequence was BLAST-searched against homologous sequences. The 564 bp had 99 to 100% homology with *P. neglecta* MH860161.1, AY682935.1, KP689121.1, and MG572407.1, according to the BLASTn analysis. The outcomes of ITS identification were consistent with those obtained from morphological and microscopic features examination. Further, high similarity sequences were selected, and MEGA 7.0 was used to build a phylogenetic tree based on the maximum likelihood method (Figure 1). The isolate is closely related to cluster of *P. neglecta* according to the BLAST analyses.

### 3.6. Bioactivity Analysis

#### 3.6.1. Antioxidant

**Figure 2:**
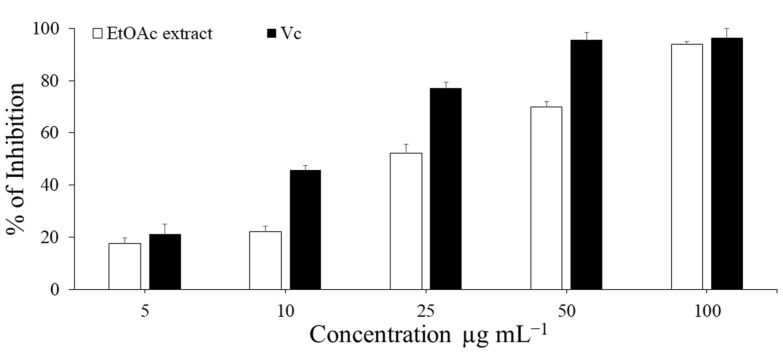
DPPH radical scavenging activity of EtOAc extract of *P. neglecta* the standard antioxidant (Vc) at different concentrations (5–100 μg mL^−1^). The IC_50_ values of Vc and EtOAc extract were determined from the equations by y= 0.6873x + 41.041 and y= 0.7937x + 20.921, respectively. Each value is expressed as mean ±SD (*n* = 3).

As shown in (Figure 2), *P. neglecta* EtOAc extract demonstrated considerable dose dependent free radical scavenging activity. Positive control; Vc revealed notable antioxidant capacity of 96.4% at a dose of 100 μg mL^−1^, whereas fungal extract showed outstanding activities (17.5–93.9%) at doses ranging from 5 to 100 μg mL^−1^. On the other side, EtOAc extract and Vc had remarkable calculated IC_50_ values of 36.6 and 13.1 μg mL^−1^, respectively. According to the findings, *P. neglecta* might be a promising source of natural antioxidants.

#### 3.6.2. Cytotoxic Activity

**Figure 3:**
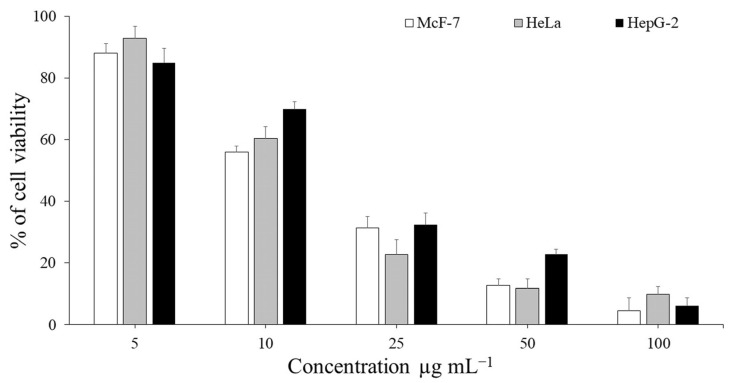
The in vitro cytotoxicity of EtOAc extract of *P. neglecta* on the viability of McF-7, HeLa and HepG-2 cell lines. Tumor cells were treated with different concentrations ranged of (5–100 μg mL^−1^), and the cell viability was evaluated by MTT assay. The data represent the IC_50_ values of 22.4, 23.2 and 28.9, respectively. The bars on the graph represent mean ±SD as a percentage of proliferation of triplicate independent experiments (*n* = 3).

Figure 3 illustrates the MTT experiment used to assess the anti-proliferative effects of *P. neglecta* EtOAc extract on MCF-7 (breast carcinoma), HeLa (cervical carcinoma) and HepG-2 (liver carcinoma) cell lines at various concentrations ranging from 5 to 100 μg mL^−1^. All of the evaluated cancer cell lines showed dose dependence for the cytotoxic activity. In our investigation, the cytotoxicity of the *P. neglecta* EtOAc extract against the MCF-7, HeLa, and HepG-2 cell lines was considerable, with values of 4.5, 9.8, and 6.2, respectively, at dose of 100 μg mL^−1^. To our delights, The IC_50_ values on MCF-7, HeLa and HepG-2 were 22.4, 23.2 and 28.9 μg mL^−1^, respectively. Our findings imply that the *P. neglecta* residing in the leaves of *Z. spina-christi* has the ability to prevent the proliferation and viability of MCF-7, HeLa and HepG-2 cell lines.

### 3.7. DNA Protection Ability

**Figure 4:**
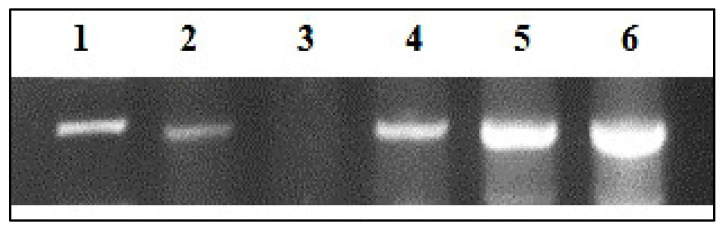
DNA damage protection by endophytic fungal EtOAc extract of *P. neglecta.* Lane 1: native λ DNA, Lane 2: λ DNA + Fenton’s reagent + quercetin; positive control, Lane 3: λ DNA + Fenton’s reagent, Lane 4: λ DNA + EF2 (200 μg) Fenton’s reagent + Extract (0.1 μg mL^−1^), Lane 5: λ DNA + Fenton’s reagent + Extract (1.0 μg mL^−1^), Lane 6: λ DNA + Fenton’s reagent + Extract (10 μg mL^−1^).

*P. neglecta* EtOAc extract was investigated for its capacity to protect λ-DNA from oxidative damage. (Figure 4) displayed the DNA electrophoric pattern in the presence and absence of various extract doses. The generation of hydroxyl radicals by Fenton’s reaction resulted in the complete disappearance of the DNA band, totally degrading the DNA; lane 3 compared to control; lane 1. The appearance of visible bands in lanes 4, 5, and 6 at concentrations of 0.1, 1.0, and 10 μg mL^−1^ indicates that all extract doses effectively reduced oxidative stress and shielded the DNA from OH radicals produced by Fenton’s reaction. The best correlation between extract concentration and DNA protective effect can be seen in the band’s sharpness; a higher band intensity demonstrated the maximum level of activity, whilst the faint band represents the minimum level of DNA damage prevention. The highest DNA damage protective activity among fungal extract concentrations was recorded at 10 μg mL^−1^ (lane 6), whereas the lowest performance was found at 0.1 μg mL^−1^ (lane 4). As a positive control, standard quercetin was employed (lane 2). The findings of this investigation suggest some degree of DNA protection from OH radical damage provided by *P. neglecta* EtOAc extract.

### 3.8. FT-IR

**Figure 5:**
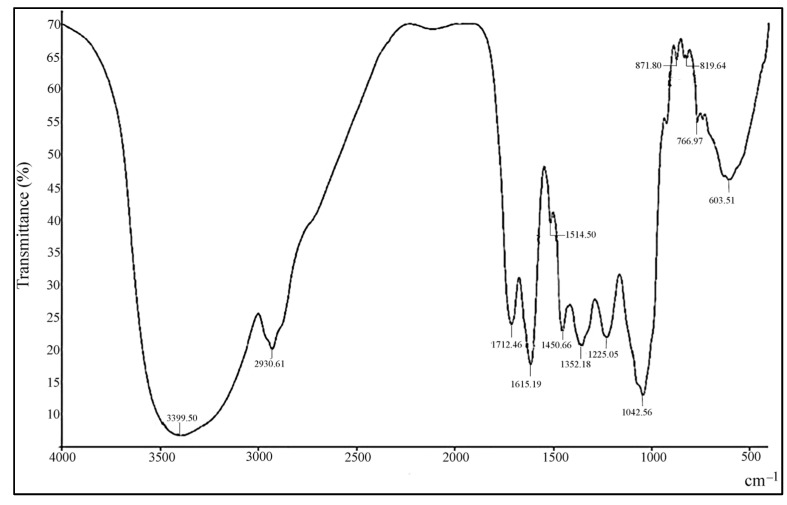
Phytochemical screening by FTIR spectroscopic analysis of *P. neglecta* EtOAc extract.

In the current study, FTIR analysis was used to identify potential functional groups based on peak values using the EtOAc extract of the endophytic fungus *P. neglecta*. The following peaks with intensities (cm^−1^) of 3399.50 (N–H, aliphatic primary amine or phenol, OH group), 2930.61 (C–H, Alkane), 1712.46 (C=O, carbonyl/ketone), 1615.19 (C=C, unsaturated ketone or N–H bend), 1514.50 (N–O, Nitro group), 1450.66 (C–H, Alkane), 1352.18 (S=O, sulfonamide or aromatic amines), 1225.05 (C–O, alkyl ether), 1042.56 (–O–, esters or O–H, carboxylic acid), 871.80 (C–H, 1,3 di-substituted), 819.64 (C=C, alkene), 766.97 (C–H, 1,2 di-substituted) and 603.51 (C–Br, alkyl halides) (Figure 5). The peaks that were detected show the existence of several functional groups, which are predisposed to the presence of bioactive compounds.

### 3.9. Detection of Bioactive Compounds by GC-MS Analysis

**Figure 6:**
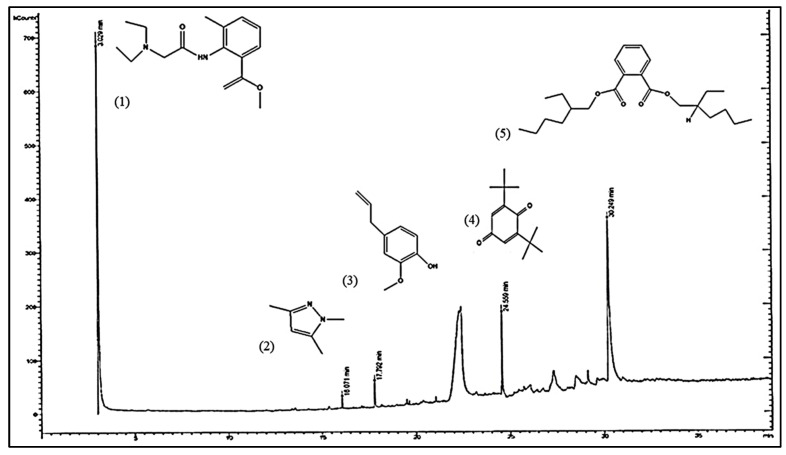
GC-MS chromatogram of EtOAc extract of *P. neglecta.* Chemical structures of bioactive compounds identified as: (1) tolycaine; (2) 1H-pyrazol, 1,3,5-trimethyl-; (3) eugenol; (4) 2,5-cyclohexadiene-1,4-dione, 2,6-bis(1,1-dimethyl)-; (5) bis(2-ethylhexyl) phthalate.

Additionally, GC-MS analysis was performed on *P. neglecta* EtOAc extract to identify metabolites profile (Figure 6). Depending on the NIST library, multiple peaks of active compounds were resolved and shown in (Table 4) as well as with their retention time (RT), molecular formula, molecular weight, and peak area. The chromatogram showed that tolycaine, 1H-pyrazol, 1,3,5-trimethyl-, eugenol, 2,5-cyclohexadiene-1,4-dione, 2,6-bis(1,1-dimethyl)- and bis(2-ethylhexyl) phthalate were the five major active compounds of *P. neglecta*. The detection of these metabolites demonstrated the capacity of endophytic fungus *P. neglecta* to create compounds that closely reflect its biological bioactivities.

## 4. Discussion

Throughout the world, endophytes have been extensively investigated in a variety of unexplored environments. Since endophytes are chemical synthesizers found inside plants, it is crucial to unlock the potential of this untapped resource from indigenous plants [34]. Healthy plants possess microorganisms called endophytes that defend them from infections and pests. Endophytes play an important role as biocatalysts in the biocatalytic process in the pharmaceutical sector [35]. All plant species had endophytic fungi, which were examined for endophytic microbial components [36]. The variety of endophytic fungi correlated with *Z. spina* was assessed in this investigation, which was conducted in Al-Ahsa Oasis, Saudi Arabia. Since leaves are rich and plentiful source of fungal endophytes, they were selected for this study’s analysis of their populations [37].

In the current investigation, 450 leaf fragments of *Z. spina* yielded a total of 191 fungal endophytic isolates, resulting in an overall colonization rate of 50.3%. Herein, based on morphological characteristics, the isolates were recovered and categorized into 11 genera, representing 26 distinct species. It is noteworthy that endophytic fungi that have previously been reported as common endophytes in other plants, including *Alternaria* sp., *Aspergillus* sp., *Chaetomium* sp., *Fusarium* sp., *Pestalotiopsis* sp., and *Xylaria* sp., were recognized from the recovered isolates by their morphological features [38,39,40,41,42,43]. Here, *A. flavus*, *A. cyanophagus*, *C. globosum*, and *P. neglecta* were the most prevalent fungi, with colonization rates ranging from 4.2% to 7.6%.

The fact that Saudi Arabia is a desert nation may be a contributing factor to the study’s relatively low overall colonization rate. It is noteworthy that most of the recovered fungal taxa in this study are Ascomycota. This is consistent with several studies that have demonstrated that the endophytic fungi affiliated to medicinal plants were mainly Ascomycetes [32,44,45].

Endophytic fungi are gaining popularity among researchers as an alternate source for eradicating human and plant infections [46]. It is interesting to note that the rise in pathogenic fungi with various drug-resistant patterns has been noticed, making the development of novel antifungal drugs an urgent demand in order to research new medications and pharmaceuticals [47]. According to the results of the present investigation, the endophytic fungus *P. neglecta* has the capacity to prevent the development of the pathogenic test fungi. This capability may be related to its ability to generate secondary metabolites possessing growth inhibitory activity. This is consistent with findings of [48], who reported that *P. neglecta* could be served as antifungal agent. However, a previous study that suggested that endophytic fungi might be a good source of bioactive compounds supports our findings [31, 49, 50, 5151]. Furthermore, I want to draw attention to the moderate to weak inhibition of pathogenic fungal growth that isolates of *C. globosum, A. flavus* and *A. cyanophagus* exhibited. This is evidence that the activity spectra of endophytes varied substantially between isolates, indicating that a variety of biological active molecules involved in the antifungal action. This is in agreement with Khan [52], who found bioactive metabolites with anti-pathogenic in *C. globosum*, *A. cyanophagus* and *A. flavus*.

Comparatively to other fungal crude extracts, the phytochemical preliminary investigation of the crude extract of *P. neglecta* showed the presence of different fungal metabolites, including phenols, flavonoids, terpenoids, alkaloids, tannins, steroids, and saponins. This concurs a report by Gopiesh and Kannabiran [53] that many phytochemicals have been detected in the endophytes. Since, endophytes have been reported to be capable of producing particular metabolites but not others [54], our findings are supported. Phenols and terpenes have primary and secondary antioxidant activity [55,56]. Moreover, bioactivity is provided by phenol, alkaloids, flavonoid, tannin, and terpenoids [57,58].

After developing the strategy, *P. neglecta*, a potential endophytic fungus, has been successfully identified using molecular techniques [59]. Phylogenetic importance of morphological characteristics was discussed by Wei [60]. It was suggested that morphological traits rather than host association should be considered when describing new *Pestalotiopsis* species, and that molecular phylogenetic data is also required to demonstrate that the taxon is distinct from existing recognized species. *Pestalotiopsis* is thought to be a pathogen that causes leaf spots, whilst some research have found that certain species of *Pestalotiopsis* may also operate as antifungal agents, as well as producers of bioactive substances [48,61].

Here, we select ethyl acetate extraction since it is the most effective approach for isolating fungal secondary metabolites [62]. As an extraction solvent, ethyl acetate preferentially extracts high molecular weight polyphenols and low molecular weight phenolic compounds [63].

The most widely used approach to evaluate the antioxidant activity, or free radical scavenging abilities of antioxidant compounds in plants and extracts is DPPH radical scavenging activity. The assay is based on DPPH’s propensity to decolorize in the presence of antioxidants; DPPH is a stable free radical. An odd electron in the DPPH radical induces the absorbance at 517 nm. When an antioxidant compound donates an electron to DPPH, the absorbance values decrease, which may be quantitatively measured by keeping track of the change in absorbance [64]. Antioxidants are recognized to offer defense against a wide range of diseases. This is why there is such a high level of interest in natural antioxidants and their significance in human nutrition and health [65]. Researchers have looked into several medicinal herbs, spices, fruits, vegetables, and fungi as prospective sources of safe natural antioxidants. Numerous fungi, endophytes, have recently been discovered to exhibit antioxidant activity [66]. For that, they are known to produce a variety of novel metabolites with antioxidant activity and are as potent as synthetic antioxidants, endophytic fungi such as *Cladosporium* sp., *Chaetomium* sp., *Phoma* sp., *Torula* sp., and *Penicillium* have been extensively studied as sources of potentially natural antioxidants [66,67]. In this study, the EtOAc extract of *P. neglecta* showed good antioxidant activity, which may be explained by different mechanisms including the binding of transition metal ion catalysts, the avoidance of chain initiation, the decomposition of peroxides, and the scavenging of free radicals [68]. Our findings are interestingly in line with a literature review that found that the crude extract of *P. neglecta* showed antioxidant activity with 59% and 62.1% inhibition in DPPH free radical scavenging activity [69]. To our delights, the IC_50_ value recorded in this investigation (36.6 µg mL^−1^) was considerably lower than that of the endophytic fungi *Neopestalotiopsis protearum*, and *Cytospora rhizophorae* which displayed effective antiradical activity with IC_50_ values of 1240 and 330 µg mL^−1^, respectively [70]. 

The assessment of natural product cytotoxicity is the initial stage of the development of anticancer drugs [59]. It has been documented in the literature that endophytes can produce bioactive substances with therapeutic value or potential [71,72,73], and it is conceivable that the endophytic fungi may be the original source of these metabolites [21,74]. According to the current investigation, *P. neglecta* crude extract shown remarkable cytotoxicity against MCF-7, HeLa 7 and HepG-2 cell lines, with IC_50_ values of 22.4, 23.2, and 28.9 µg mL^−1^, respectively, which are lower than the National Cancer Institute (NCI) standard. The NCI guidelines state that a prospective anticancer agent is any crude extract with an IC_50_ value less than 30 µg mL^−1^ [75]. Moreover, several studies have been conducted on endophytes in an effort to find powerful and cutting-edge medications for the treatment of cancer [32,76,77]. Our current research will be valuable in identifying new active natural products from *P. neglecta* that could be found to be newer cytotoxic drugs.

Free radicals have the potential to disrupt DNA strands, which ultimately aids in the development of mutagenesis and cytotoxicity. Hydroxyl radicals react with DNA via base addition or hydrogen atoms abstraction from the sugar moiety. According to Lee et al. [78], Fenton’s can generate highly reactive OH via Fenton reaction since they are composed of H_2_O_2_, Fe^3+^ and ascorbic acids.

The EtOAc extract of *P. neglecta* was tested for its ability to prevent Fenton-induced DNA damage. In the current study, 10 µg mL^−1^ was effective in preventing λ DNA damage from OH radicals caused by Fenton. Despite the fact that the EtOAc extract had relevant cytotoxic activity against MCF-7, HeLa and HepG-2 cell lines. It also demonstrated outstanding antioxidant activity. Hence, this contradicting pattern can therefore be attributed to the *P. neglecta* extract’s existence of different phytochemicals, which exhibit multifunction biological activity. This is the first report to validate DNA damage protection by *P. neglecta*. Undeniably, *Pestalotiopsis* species received attention due to their capacity to produce numerous structurally bioactive secondary metabolites. Some of them may be crucial as potential pharmacological targets in the future for the treatment of human diseases, the prevention of plant diseases, and the development of anticancer, antioxidants agents [79].

In light of this, we are curious to learn more about these bioactive compounds. So, we explored the metabolites derived from the powerful endophytic fungus *P. neglecta* using FT-IR and GC-MS analyses. According to the spectra at different wavelengths, FTIR spectroscopic examination of EtOAc extract revealed the presence of different functional groups. The ester functional group, which is known to be δ-lactones, is present among other functional groups. This compound class has antitumor properties [80]. Additionally, the presence of phenol, alkenes, amines, carboxylic acid, and alkyl halides functional groups in the EtOAc extract was the reason of its potential antimicrobial and therapeutic property [81,82]. The existence of various functional groups highlights the secondary metabolites’ complex structure.

Further, this encourages us to use GC-MS analyses to characterize the metabolites. Based on the compounds’ peak area, RT, molecular weight, and molecular formula, they were determined. The NIST database and the relevant published literature were used to interpret the mass spectrum. Tolycaine, 1H-pyrazol, 1,3,5-trimethyl-, eugenol, 2,5-cyclohexadiene-1,4-dione, 2,6-bis(1,1-dimethyl)- and bis(2-ethylhexyl) phthalate were identified to be predominant compounds produced by *P. neglecta*. The literature that is currently available has revealed that these compounds exhibit different bioactive characteristics. According to reports, pyrazol has potent antiviral, antibacterial, antifungal, anticancer and antioxidant properties [83,84]. Another compound, eugenol, has received much attention because it possesses a wide range of biological activities, including antimicrobial [80], antifungal [85,86,87,88], antioxidant [89] and anti-carcinogenic [90]. In addition, the potent antifungal compound 2,5-cyclohexadiene-1,4-dione, 2,6-bis(1,1-dimethyl)- [91]. Moreover, phthalate showed powerful antimicrobial activity as well as strong cytotoxicity against MCF-7, HeLa and HepG-2 [92,93]. Furthermore, the nitrogen compound tolycaine, which has good antimicrobial properties, was detected [94]. It should be mentioned that our fungal extract shown considerable in vitro antifungal, cytotoxic, and antiradical activities. Therefore, the presence of these relevant compounds may be responsible for such activities. To the best of our knowledge, this is the first report to record these bioactive compounds derived from *P. neglecta*. Since taxol, isopestacin, and pestacin were previously reported drugs that originate from *Pestalotiopsis* sp. [95,96]. Hence, the endophytic fungus *P. neglecta* studied here could provide new resources for producing metabolites of interest in biotechnological applications.

## 5. Conclusions

Most endophytic fungi have not been extensively studied in the search for natural products. Only a few plants have been studied thus far in terms of endophytic biodiversity and potential bioactive secondary metabolite production. This is probably the first study that demonstrates the richness of endophytic fungal communities in the healthy leaf tissues of *Z. spina-christi*, collected from Al Ahsa Oasis, Saudi Arabia. The successful colonization of this plant by such fungi suggests that they could be used in future antifungal drug development. From recovered fungal endophyte, one isolate was identified as *P. neglecta*, found to be producers of phenols, flavonoids, terpenoids, alkaloids, tannins, steroids, and saponins; additionally, its extract shown relevant antioxidant and cytotoxic activities as well as successfully protecting the DNA from free radical damage. Moreover, we conducted a GC-MS analysis to determine bioactive compounds, which were produced by *P. neglecta*. Such findings offer a promising opportunity for the development of drugs from medicinal plant-associated endophytic fungi. Therefore, further research is needed in the demand for fractionation of these bioactive products to investigate the mechanism of *P. neglecta* metabolites with diverse biological activities. After purification and identification of the bioactive components, this crude fungal extract has the potential to be an intriguing source for novel pharmacological drugs. Despite the fact that these compounds are produced in small amounts in nature, this promising endophyte can nevertheless be used to accelerate growth and amplification by isolation, genetic modification, and industrial scale-up. Furthermore, using endophytes as alternate source of bioactive metabolites prevents the host plant from being overused, protecting biodiversity.

## Figures and Tables

**Table 1:** Isolation, colonialization and dominance frequency of endophytic fungi from plant leaf of *Z. spina-christi*.

Endophytic Fungi			No.	*CF%	*DF%
Genus	Species	Isolate Code			
*Penicillium*			24		
	*P. chrysogenum*	YH-1	13	3.4	6.8
	*P. citrinum*	YH-2	7	1.8	3.6
	*P. corylophilum*	YH-3	4	1.1	2.2
*Chaetomium*			23		
	*C. globosum*	YH-4	19	5.0	9.9
	*C. gracile*	YH-5	2	0.5	1.0
	*C. elatum*	YH-6	2	0.5	1.0
*Aspergillus*			28		
	*A. versicolor*	YH-7	4	1.1	2.2
	*A. terreus*	YH-8	1	0.3	0.6
	*A. ochraceus*	YH-9	7	1.8	3.6
	*A. flavus*	YH-10	16	4.2	8.3
*Colletotrichum*			9		
	*C. magnum*	YH-11	6	1.6	3.2
	*C. gloeosporioides*	YH-12	3	0.8	1.6
*Periconia*			8		
	*P. macrospinosa*	YH-13	8	2.1	4.2
*Xylaria*			12		
	*X. bambusicola*	YH-14	3	0.8	0.6
	*X. hypoxylon*	YH-15	9	2.4	4.8
*Acremonium*			18		
	*A. cyanophagus*	YH-16	18	4.7	9.3
*Alternaria*			5		
	*Alt. alternata*	YH-17	4	1.1	2.2
	*Alt. tenuissima*	YH-18	1	0.3	0.6
*Fusarium*			19		
	*F. oxysporum*	YH-19	13	3.4	6.8
	*F. subglutinans*	YH-20	3	0.8	1.6
	*F. culmorum* *F. solani*	YH-21YH-22	12	0.30.5	0.61.0
*Cladosporium*			16		
	*C. tenuissimum*	YH-23	2	0.5	1.0
	*C. nigrellam*	YH-24	2	0.5	1.0
	*C. cladosporioides*	YH-25	12	3.2	6.4
*Pestalotiopsis*			29		
	*P. neglecta*	YH-26	29	7.6	15.1
Total			191	50.3	

*CF%, colonialization frequency percentage; *DF%, fungal dominance frequency percentage.

**Table 2:** Antifungal activity of endophytic fungi isolated from *Z. spina-christi* against pathogenic fungi according to the dual culture.

Pathogenic Fungi	Fungal Endophytes			
	*P. neglecta*	*A. cyanophagus*	*A. flavus*	*C. globosum*
*Alt. alternata*	+++	−	+	++
*C. albicans*	++	+	++	+
*F. oxysporum*	+++	+++	−	+
*B. cinerea*	+++	−	+++	+++
*P. ultimum*	+++	++	++	+

The antifungal activity is expressed by the diameter of inhibition zone; >10 mm (strong inhibition, +++); 2–10 mm (moderate inhibition, ++); <2 mm (weak inhibition, +); not active (−, no inhibition).

**Table 3:** Phytochemical activity screening of crude extracts from endophytic fungi isolated from *Z. spina-christi*.

Endophytic Fungi	Alkaloids	Phenols	Flavonoids	Saponins	Steroids	Terpenoids	Tannins
*P. neglecta*	+	+	+	+	+	+	+
*A. cyanophagus*	+	−	−	+	+	−	−
*A. flavus*	−	+	+	−	+	−	+
*C. globosum*	+	+	−	−	−	+	+

(+), mean present; (−) mean absent.

**Table 4:** GC-MS analysis of *P. neglecta* endophytic EtOAc extract. The possible existence of bioactive compounds and its retention time, peak area, molecular formula, and molecular weight.

S. no.	Retention Time	Peak Area	Compound Name (IUPAC)	Molecular Formula	Molecular Weight
1	3.029	2.458	Tolycaine	C_15_H_22_N_2_O_3_	278
2	16.071	56371	1H-Pyrazol, 1,3,5-trimethyl-	C_6_H_10_N_2_	110
3	17.792	99222	Eugenol	C_10_H_12_O_2_	164
4	24.559	383248	2,5-Cyclohexadiene-1,4-dione, 2,6-bis(1,1-dimethyl)-	C_14_H_20_O_2_	220
5	30.249	1.662	Bis(2-ethylhexyl) phthalate	C_24_H_38_O_4_	390

## Data Availability

The data that support the findings of this study are available from the corresponding author upon reasonable request.

## References

[1] Aladesanmi A.J., Iwalewa E.O., Adebajo A.C., Akinkunmi E.O., Taiwo B.J., Olorunmola F.O., Lamikanra A. (2006). Antimicrobial and antioxidant activities of some Nigerian medicinal plants. Afr. J. Tradit. Complement. Altern. Med..

[2] Bérdy J. (2005). Bioactive microbial metabolites. J. Antibiot..

[3] Sun Y., Wang Q., Lu X., Okane I., Kakishima M. (2011). Endophytic fungal community in stems and leaves of plants from desert areas in China. Mycol. Prog..

[4] Liu C., Liu T., Yuan F., Gu Y. (2010). Isolating endophytic fungi from evergreen plants and determining their antifungal activities. Afr. J. Microbiol. Res..

[5] Meena K.K., Sorty A.M., Bitla U.M., Meena K.K., Sorty A.M., Bitla U.M., Choudhary M., Gupta P., Pareek A., Singh D.P. (2017). Abiotic stress responses and microbe-mediated mitigation in plants: The omics strategies. Front. Plant Sci..

[6] Alhakmani F., Khan S.A., Ahmad A. (2014). Determination of total phenol, in-vitro antioxidant and anti-inflammatory activity of seeds and fruits of *Zizyphus spina christi* grown in Oman. Asian Pac. J. Trop. Biomed..

[7] Wise R. (2008). The worldwide threat of antimicrobial resistance. Curr. Sci..

[8] Khalil A.M.A., Abdelaziz A.M., Khaleil M.M., Hashem A.H. (2021). Fungal endophytes from leaves of *Avicennia marina* growing in semi-arid environment as a promising source for bioactive compounds. Lett. Appl. Microbiol..

[9] Sharaf M.H., Abdelaziz A.M., Kalaba M.H., Radwan A.A., Hashem A.H. (2022). Antimicrobial, antioxidant, cytotoxic, activities and phytochemical analysis of fungal endophytes isolated from *Ocimum Basilicum*. Appl. Biochem. Biotechnol..

[10] Maharachchikumbura S.S.N., Guo L.D., Chukeatirote E., Bahkali A.H., Hyde K.D. (2011). *Pestalotiopsis* morphology, phylogeny, biochemistry and diversity. Fungal Divers..

[11] Sharma D., Pramanik A., Agrawal P.K. (2016). Evaluation of bioactive secondary metabolites from endophytic fungus *Pestalotiopsis neglecta* BAB-5510 isolated from leaves of *Cupressus torulosa* D.Don. 3 Biotech.

[12] Kumaran R.S., Kim H.J., Hur B.K. (2010). Taxol-producing [corrected] fungal endophyte, *Pestalotiopsis* species isolated from *Taxus cuspidate*. J. Biosci. Bioeng..

[13] Deyrup S.T., Swenson D.C., Gloer J.B., Wicklow D.T. (2006). Caryophyllene sesquiterpenoids from a fungicolous isolate of *Pestalotiopsis disseminata*. J. Nat. Prod..

[14] Ding G., Liu S., Guo L., Zhou Y., Che Y. (2008). Antifungal metabolites from the plant endophytic fungus *Pestalotiopsis foedan*. J. Nat. Prod..

[15] Elsabea A.M.R. (2012). An economic study of processing problems for the main important varieties of dates in Saudi Arabia. Ann. Agric. Sci..

[16] Almadini A.M., Ismail A.I.H., Ameen F.A. (2021). Assessment of farmers practices to date palm soil fertilization and its impact on productivity at Al-Hassa oasis of KSA. Saudi J. Biol. Sci..

[17] Musselman L.J. (1995). Handbook of arabian medicinal plants. Shahina A. Ghazanfar. Econ. Bot..

[18] Hawar S.N. (2022). Extracellular enzyme of endophytic fungi isolated from *Ziziphus spina* leaves as medicinal plant. Int. J. Biomater..

[19] Dobranic J.K., Johnson J.A., Alikhan Q.R. (1995). Isolation of endophytic fungi from eastern larch (*Larix lancina*) leaves from New Brunswick, Canada. Can. J. Microbiol..

[20] Barnett H.L., Hunter B.B. (2006). Illustrated Genera of Imperfect Fungi.

[21] Strobel G., Daisy B., Castillo U., Harper J. (2004). Natural products from endophytic microorganisms. J. Nat. Prod..

[22] Seifert K.A. (2008). Compendium of soil fungi-by Domsch, K.H.; Gams, W.; Anderson, T.H. Eur. J. Soil Sci..

[23] Verma V.C., Kharwar R.N., Strobel G.A. (2009). Chemical and functional diversity of natural products from plant associated endophytic fungi. Nat. Prod. Commun..

[24] Hata K., Futai K. (1995). Endophytic fungi associated with healthy pine needles and needles infested by the pine needle gall midge, *Thecodiplosis japonensis*. Canad. J. Bot..

[25] Kumar D.S.S., Hyde K.D. (2004). Biodiversity and tissue recurrence of endophytic fungi in *Tripterygium wilfordii*. Fungal Divers..

[26] Lu L., Karunarathna S.C., Hyde K.D., Suwannarach N., Elgorban A.M., Stephenson S.L., Al-Rejaie S., Jayawardena R.S., Tibpromma S. (2022). Endophytic fungi associated with coffee leaves in China exhibited in vitro antagonism against fungal and bacterial pathogens. J. Fungi.

[27] Kokate C.K., Purohit A.P., Gokhale S.B. (2005). Pharmacognosy.

[28] Maobe M.A.G., Gatebe E., Gitu L., Rotich H. (2013). Preliminary phytochemical screening of eight selected medicinal herbs used for the treatment of diabetes, malaria and pneumonia in Kisii region, Southwest Kenya. Eur. J. Appl. Sci..

[29] Plaza G.A., Upchurch R., Brigmon R.L., Whitman W.B., Ulfig K. (2004). Rapid DNA extraction for screening soil filamentous fungi using PCR amplification. Pol. J. Environ. Stud..

[30] White T.J., Bruns T., Lee S., Taylor J., Innis M.A., Gelfand D.H., Sninsky J.J., White T.J. (1990). Amplification and direct sequencing of fungal ribosomal RNA genes for phylogenetics. PCR Protocols: A Guide to Methods and Applications.

[31] Yehia R.S. (2022). Evaluation of the biological activities of β-glucan isolated from *Lentinula edodes*. Lett. Appl. Microbiol..

[32] Mahnashi M.H., Alqahtani Y.S., Alyami B.A., Alqarni A.O., Ullah F., Wadood A., Sadiq A., Shareef A., Ayaz M. (2021). Cytotoxicity, anti-angiogenic, anti-tumor and molecular docking studies on phytochemicals isolated from *Polygonum hydropiper* L.. BMC Complement. Med. Ther..

[33] Ghanta S., Banerjee A., Poddar A., Chattopadhyay S. (2007). Oxidative DNA damage preventive activity and antioxidant potential of *Stevia rebaudiana* (Bertoni) Bertoni, a natural sweetener. J. Agric. Food Chem..

[34] Dar R.A., Rather S.A., Mushtaq S., Qazi P.H. (2015). Purification and characterization of endophytic fungal strains from four different high value medicinal plants of Kashmir valley. Int. J. Phytopharm..

[35] Boyle C., Gotz M., Dammann-Tugend U., Schulz B. (2001). Endophyte-host interaction III. Local vs. Systemic colonization. Symbiosis.

[36] Carroll M.C. (2004). The complement system in regulation of adaptive immunity. Nat. Immunol..

[37] Arnold A.E., Maynard Z., Gilbert G.S., Coley P.D., Kursar T.A. (2000). Are tropical fungal endophytes hyperdiverse?. Ecol. Lett..

[38] Davis E.C., Franklin J.B., Shaw A.J., Vilgalys R. (2003). Endophytic *Xylaria* (Xylariaceae) among liverworts and angiosperms: Phylogenetics, distribution, and symbiosis. Am. J. Bot..

[39] Demers J.E., Gugino B.K., Jiménez-Gasco M.M. (2015). Highly diverse endophytic and soil *Fusarium oxysporum* populations associated with field-grown tomato plants. Appl. Environ. Microbiol..

[40] Kjer J., Wray V., Edrada-Ebel R., Ebel R., Pretsch A., Lin W., Proksch P. (2009). Xanalteric acids I and II and related phenolic compounds from an endophytic *Alternaria* sp. isolated from the mangrove plant Sonneratia alba. J. Nat. Prod..

[41] Liu Y., Chen S., Liu Z., Lu Y., Xia G., Liu H., He L., She Z. (2015). Bioactive metabolites from mangrove endophytic fungus *Aspergillus* sp. 16-5B. Mar. Drugs.

[42] Yang X.L., Huang L., Li H.Y., Yang D.F., Li Z.Z. (2015). Two new compounds from the plant endophytic fungus *Pestalotiopsis versicolor*. J. Asian Nat. Prod. Res..

[43] Zhang G., Zhang Y., Qin J., Qu X., Liu J., Li X., Pan H. (2013). Antifungal metabolites produced by *Chaetomium globosum* No. 04, an endophytic fungus isolated from *Ginkgo biloba*. Ind. J. Microbiol..

[44] Bhardwaj A., Sharma D., Jodan N., Agrawal P.K. (2015). Antimicrobial and phytochemical screening of endophytic fungi isolated from spikes of *Pinus rouxburghii*. Arch. Clin. Microbiol..

[45] Tan X.M., Zhou Y.Q., Zhou X.L., Xia X.H., Wei Y., He L.L., Tang H.Z., Yu Y. (2018). Diversity and bioactive potential of culturable fungal endophytes of *Dysosma versipellis*; a rare medicinal plant endemic to China. Sci. Rep..

[46] Gao F.K., Dai C.C., Liu X.Z. (2010). Mechanisms of fungal endophytes in plant protection against pathogens. Afr. J. Microbiol. Res..

[47] Muhaj F.F., George S.J., Nguyen C.D., Tyring S.K. (2022). Antimicrobials and resistance part II: Antifungals, antivirals, and antiparasitics. J. Am. Acad. Dermatol..

[48] Tejesvi M.V., Kini K.R., Prakash H.S., Subbiah V., Shetty H.S. (2007). Genetic diversity and antifungal activity of species of *Pestalotiopsis* isolated as endophytes from medicinal plants. Fungal Divers..

[49] Phongpaichit S., Rungjindamai N., Rukachaisirikul V., Sakayaroj J. (2006). Antimicrobial activity in cultures of endophytic fungi isolated from *Garcinia* species. FEMS Immunol. Med. Microbiol..

[50] Suryanarayanan T.S. (2019). Repository of fungal endophytes at Vinstrom, Chennai: Waiting to be harnessed. Curr. Sci..

[51] Sudha V., Govindaraj R., Baskar K., Al-Dhabi N.A., Duraipandiyan V. (2016). Biological properties of endophytic fungi. Braz. Arch. Biol. Technol..

[52] Khan M.S., Gao J., Munir I., Zhang M., Liu Y., Moe T.S., Xue J., Zhang X. (2021). Characterization of endophytic fungi, *Acremonium* sp., from *Lilium davidii* and analysis of its antifungal and plant growth-promoting effects. Biomed. Res. Int..

[53] Gopiesh k.V., Kannabiran K. (2008). Antimicrobial activity of saponin fractions of the leaves of *Gymnema sylvestre* and *Eclipta prostrata*. World J. Microbiol Biotechnol..

[54] Selim S.A., El-Alfy S., Al-Ruwaili M., Abdo A., Al-Jaouni S. (2011). Susceptibility of imipenem-resistant *Pseudomonas* aeruginosa to flavonoid glycosides of date palm (*Phoenix dactylifera* L.) tamar growing in Al Madinah, Saudi Arabia. Afr. J. Biotechnol..

[55] Gülçin I. (2006). Antioxidant and antiradical activities of L-carnitine. Life Sci..

[56] Hajdú Z., Hohmann J., Forgo P., Martinek T., Dervarics M., Zupkó I., Falkay G., Cossuta D., Máthé I. (2007). Diterpenoids and flavonoids from the fruits of *Vitex agnus-castus* and antioxidant activity of the fruit extracts and their constituents. Phytother. Res..

[57] Tran H.B.Q., Mcrae J.M., Lynch F., Palombo E.A., Méndez-Vilas A. (2010). Identification and bioactive properties of endophytic fungi isolated from phyllodes of *Acacia* species. Current Research, Technology and Education Topics in Applied Microbiology and Microbial Biotechnology.

[58] Muthukrishnan S.D., Subramaniyan A. (2012). Phytochemical constituents of *Gloriosa superb* seed, tuber and leaves. Res. J. Pharm. Biol. Chem. Sci..

[59] Devi N.N., Prabakaran J.J., Wahab F. (2012). Phytochemical analysis and enzyme analysis of endophytic fungi from *Centella asiatica*. Asian Pac. J. Trop. Med..

[60] Wei J.G., Tong X., Liang-Dong G., Liu A.R., Ying Z., Pan X.H. (2005). Endophytic *Pestalotiopsis* species from southern China. Mycosystema.

[61] Li E., Jiang L., Guo L., Zhang H., Che Y. (2008). Pestalachlorides A–C, antifungal metabolites from the plant endophytic fungus *Pestalotiopsis adusta*. Bioorg. Med. Chem..

[62] Garcia A., Rhoden S.A., Bernardi-Wenzel J., Orlandelli R.C., Azevedo J.L., Pamphile J.A. (2012). Antimicrobial activity of crude extracts of endophytic fungi isolated from medicinal plant *Sapindus saponaria* L.. J. Appl. Pharm. Sci..

[63] Scholz E., Rimpler H. (1989). Proanthocyanidins from *Krameria triandra* root. Planta Med..

[64] Sri-Harsha P.S.C., Khan M.I., Prabhakar P., Giridhar P. (2013). Cyanidin-3-glucoside, nutritionally important constituents and in vitro antioxidant activities of *Santalum album* L. berries. Int. Food Res. J..

[65] Chandra P., Arora D.S. (2009). Antioxidant activity of fungi isolated from soil of different areas of Punjab, India. J. Appl. Nat. Sci..

[66] Song T.Y., Yen G.C. (2002). Antioxidant properties of *Antrodia camphorata* in submerged culture. J. Agric. Food Chem..

[67] Gebhardt P., Dornberger K., Gollmick F.A., Gräfe U., Härtl A., Görls H., Schlegel B., Hertweck C. (2007). Quercinol, an anti-inflammatory chromene from the wood-rotting fungus *Daedalea quercina* (Oak Mazegill). Bioorg. Med. Chem. Lett..

[68] Bounatirou S., Smiti S., Miguel M.G., Faleiro L., Rejeb M.N., Neffati M., Costa M.M., Figueiredo A.C., Barroso J.G., Pedro L.G. (2007). Chemical composition, antioxidant and antibacterial activities of the essential oils isolated from Tunisian *Thymus capitatus* Hoff. et Link. Food Chem..

[69] Güder A., Korkmaz H. (2012). Evaluation of in vitro antioxidant properties of hydroalcoholic solution extracts *Urtica dioica* L., *Malva neglecta* Wallr. and their mixture. Iran J. Pharm. Res..

[70] Zhou X., Chan K., Yeung J.H. (2012). Herb-drug interactions with Danshen (*Salvia miltiorrhiza*): A review on the role of cytochrome P450 enzymes. Drug Metabol. Drug Interact..

[71] Zhao J., Zhou L., Wang J., Shan T., Zhong L., Liu X., Gao X.Y., Méndez-Vilas A. (2010). Endophytic fungi for producing bioactive compounds originally from their host plants. Current Research, Technology and education Topics in Applied Microbiology and Microbial Biotechnology.

[72] Kharwar R.N., Mishra A., Gond S.K., Stierle A., Stierle D. (2011). Anticancer compounds derived from fungal endophytes: Their importance and future challenges. Nat. Prod. Rep..

[73] Kusari S., Pandey S.P., Spiteller M. (2013). Untapped mutualistic paradigms linking host plant and endophytic fungal production of similar bioactive secondary metabolites. Phytochemistry.

[74] Chen L., Zhang Q.Y., Jia M., Qian-Liang M., Yue W., Rahman K., Lu-Ping Q., Han T. (2016). Endophytic fungi with antitumor activities: Their occurrence and anticancer compounds. Crit. Rev. Microbiol..

[75] Cui J.L., Guo S.X., Xiao P.G. (2011). Antitumor and antimicrobial activities of endophytic fungi from medicinal parts of *Aquilaria sinensis*. J. Zhejiang Univ. Sci. B.

[76] Zhan J., Burns A.M., Liu M.X., Faeth S.H., Gunatilaka A.A. (2007). Search for cell motility and angiogenesis inhibitors with potential anticancer activity: Beauvericin and other constituents of two endophytic strains of *Fusarium oxysporum*. J. Nat. Prod..

[77] Nascimento A.M., Conti R., Turatti I.C.C., Cavalcanti B.C., Costa-Lotufo L.V., Pessoa C., de Moraes M.O., Manfrim V., Toledo J.S., Toledo J.S. (2012). Bioactive extracts and chemical constituents of two endophytic strains of Fusarium oxysporum. Rev. Bras. Farmacogn..

[78] Lee J.C., Kim H.R., Kim J., Jang Y.S. (2002). Antioxidant property of an ethanol extract of the stem of *Opuntia ficus-indica* var. saboten. J. Agric. Food Chem..

[79] Singh A., Singh D.K., Kharwar R.N., White J.F., Gond S.K. (2021). Fungal endophytes as efficient sources of plant-derived bioactive compounds and their prospective applications in natural product drug discovery: Insights, avenues, and challenges. Microorganisms.

[80] Xie Y., Yang W., Tang F., Chen X., Ren L. (2015). Antibacterial activities of flavonoids: Structure-activity relationship and mechanism. Curr. Med. Chem..

[81] Ragavendran P., Sophia D., Arul Raj C., Gopalakrishnan V.K. (2011). Functional group analysis of various extracts of *Aerva lanata* (L.,) by FTIR spectrum. Pharmacologyonline.

[82] Pednekar P.A., Raman B. (2013). Antimicrobial and antioxidant potential with FTIR analysis of *Ampelocissus latifolia* (roxb.) Planch. Leaves. Asian J. Pharm. Clin. Res..

[83] Fustero S., Sánchez-Roselló M., Barrio P., Simón-Fuentes A. (2011). From 2000 to mid-2010: A fruitful decade for the synthesis of pyrazoles. Chem. Rev..

[84] Ansari A., Ali A., Asif M. (2017). Biologically active pyrazole derivatives. New J. Chem..

[85] Ali S.M., Khan A.A., Ahmed I., Musaddiq M., Ahmed K.S., Polasa H., Rao L.V., Habibullah C.M., Sechi L.A., Ahmed N. (2005). Antimicrobial activities of Eugenol and Cinnamaldehyde against the human gastric pathogen *Helicobacter pylori*. Ann. Clin. Microbiol. Antimicrob..

[86] Pinto E., Vale-Silva L., Cavaleiro C., Salgueiro L. (2009). Antifungal activity of the clove essential oil from *Syzygium aromaticum* on *Candida*, *Aspergillus* and dermatophyte species. J. Med. Microbiol..

[87] Wang C., Zhang J., Chen H., Fan Y., Shi Z. (2010). Antifungal activity of eugenol against *Botrytis cinerea*. Trop. Plant Pathol..

[88] Carrasco H., Raimondi M., Svetaz L., Liberto M.D., Rodriguez M.V., Espinoza L., Madrid A., Zacchino S. (2012). Antifungal activity of eugenol analogues. Influence of different substituents and studies on mechanism of action. Molecules.

[89] Ogata M., Hoshi M., Urano S., Endo T. (2000). Antioxidant activity of eugenol and related monomeric and dimeric compounds. Chem. Pharm. Bull..

[90] Zheng G.Q., Kenney P.M., Lam L.K. (1992). Sesquiterpenes from clove (*Eugenia caryophyllata*) as potential anticarcinogenic agents. J. Nat. Prod..

[91] Li C.W., Song R.Q., Yang L.B., Deng X. (2015). Isolation, purification, and structural identification of an antifungal compound from a *Trichoderma* strain. J. Microbiol. Biotechnol..

[92] Habib M.R., Karim M.R. (2009). Antimicrobial and cytotoxic activity of Di-(2-ethylhexyl) phthalate and anhydrosophoradiol-3-acetate isolated from *Calotropis gigantea* (Linn.) Flower. Mycobiology.

[93] Lotfy M.M., Hassan H.M., Hetta M.H., El-Gendy A.O., Mohammed R. (2018). Di-(2-ethylhexyl) Phthalate, a major bioactive metabolite with antimicrobial and cytotoxic activity isolated from River Nile derived fungus *Aspergillus awamori*. Beni-Suef Univ. J. Basic Appl. Sci..

[94] McBain A.J., Ledder R.G., Moore L.E., Catrenich C.E., Gilbert P. (2004). Effects of quaternary-ammonium-based formulations on bacterial community dynamics and antimicrobial susceptibility. Appl. Environ. Microbiol..

[95] Strobel G., Hess W., Ford E., Sidhu R., Yang X. (1996). Taxol from fungal endophytes and the issue of biodiversity. J. Ind. Microbiol..

[96] Demain A.L., Zhang L. (2005). Natural products and drug discovery. Nat. Prod..

